# Public behaviour in response to the COVID-19 pandemic: understanding the role of group processes

**DOI:** 10.1192/bjo.2020.139

**Published:** 2020-12-07

**Authors:** John Drury, Holly Carter, Evangelos Ntontis, Selin Tekin Guven

**Affiliations:** School of Psychology, University of Sussex, UK; Emergency Response Department Science and Technology, Health Protection Directorate, Public Health England, UK; School of Psychology and Health Sciences, Canterbury Christ Church University, UK; School of Psychology, University of Sussex, UK

**Keywords:** COVID-19, behaviour, groups, public health, resilience

## Abstract

**Background:**

In the absence of a vaccine, behaviour by the public is key to the response to the COVID-19 pandemic. Yet, as with other types of crises and emergencies, there have been doubts about the extent to which the public are able to engage effectively with the required behaviour. These doubts are based on outdated models of group psychology.

**Aims and argument:**

We analyse the role of group processes in the COVID-19 pandemic in three domains: recognition of threat, adherence by the public to the required public health behaviours (and the factors that increase such adherence) and actions of the many community mutual aid groups that arose during lockdown. In each case, we draw upon the accumulated research on behaviour in emergencies and disasters, as well as the latest findings in relation to the COVID-19 pandemic, to show that explanations in terms of social identity processes make better sense of the patterns of evidence than alternative explanations.

**Conclusions:**

If behaviour in the pandemic is a function of mutable group processes rather than fixed tendencies, then behavioural change is possible. There was evidence of significant change in behaviour from the public, particularly in the early days of the pandemic. Understanding the role of group processes means we can help design more effective interventions to support collective resilience in the public in the face of the pandemic and other threats. We draw out from the evidence a set of recommendations on facilitating the public response to COVID-19 by harnessing group processes.

In the absence of a vaccine, public behaviour is key to the response to the COVID-19 pandemic.^1,2^ The virus spreads through close contact between people and via surfaces, and so the actions that members of the public have taken to protect themselves and others include physical distancing (although ‘social distancing’ is the term used by many governments, the World Health Organization^3^ recommends the term ‘physical distancing’ as more accurate), regular hand-washing, working from home, wearing face coverings and self-isolating if they have symptoms. As in the case of other crises, some were doubtful about the extent to which the public would be willing and able to behave in the required ways. Often these doubts were based on the assumption that there is a generic behavioural tendency in such events, grounded in a narrowly self-interested or psychologically fragile human nature.^4^ Previous research on human responses to emergencies and disasters has indeed demonstrated some common patterns of behaviour in such events. In contrast to the most pessimistic expectations, however, collectively resilient behaviours – in particular, public mutual cooperation – are relatively common.^5^ But more importantly, this research and the resultant theory suggest the social and psychological conditions under which public behaviour in crises takes one form rather than another. Some of the most important conditions are to do with group processes, which refers to people's psychological memberships of groups or social categories and their relationship with others outside the group. Understanding behaviour in the pandemic as a function of mutable group processes, rather than fixed psychological tendencies, means a greater understanding of behavioural change – both analysing it and designing interventions to facilitate it.

The effects of a pandemic are much more dispersed in time and space than those of other emergencies, such as fires, earthquakes and terrorist attacks. Yet, like these other kinds of emergencies, a pandemic such as COVID-19 represents a mortal threat that creates collective fear. In all cases, immediate and dramatic responses are required. Therefore, in this article, as well as referring to recent research on public responses to COVID-19, we draw upon the accumulated research on behaviour in emergencies and disasters to provide insights into the role of group processes in the pandemic. Our examples of behaviour and policy in the pandemic come mostly from the UK context, as that is where the authors are based. However, we suggest that most of our points are transferable to the situation in other countries.

The starting point for a behavioural response to an emergency is the recognition of the threat,^6^ so the first part of the article will cover what we know about when and how people perceive threats. The second part will focus on adherence by the public to the required public health behaviours and the factors that increase such adherence. The third part examines the determinants of neighbourhood support and the many community mutual aid groups that arose during lockdown. Finally, we draw out from the review of evidence three recommendations on facilitating the public response to COVID-19 by harnessing group processes.

## When and how people perceive threats

‘Panic’ is a popular view of how people respond in emergencies.^7^ There are various definitions of panic, but one thing that distinguishes the concept from related notions such as fear and flight is the idea of overreaction to a perceived threat.

A basic empirical problem with the concept of panic in the context of mass emergencies is that of measurement:^8^ in an emergency, how do we know if fear is ‘excessive’? At the time, what counts as an overreaction is typically a subjective judgement. *Post hoc*, it is easier to judge, but then it is no longer a description of someone's mental state and is not explanatory.

For example, at the beginning of the COVID-19 crisis, was the extra shopping that some people carried out – so-called ‘panic buying’ – really excessive? On what criteria? If people believe (a) that they may have to stay home for prolonged periods of time, or (b) that other people will soon empty the shelves, or both (a) and (b), it is logical to buy additional goods. It may be excessive from the point of view of the observer, but psychologically it might be an understandable reaction to a perceived threat.

The panic concept is sometimes used to claim that public overreaction to signals of danger is a major cause of death in emergencies.^9^ But most sources suggest that people are more likely to die from ignoring or reacting too slowly to indications of danger than they are from overreacting.^6^ Indeed, public underreaction to signals of threat in emergencies and disasters is common.^10^ This pattern of response has sometimes been characterised as evidence of an ‘optimistic bias’.^11^ But there are a number of group-level psychological factors that determine the extent to which people respond to (versus discount) signals of impending emergency.

The first factor is the historical context. For example, if there has not been an earthquake locally in 100 years, people may not see the point in engaging in preparedness. But when they have had experience of quakes and other disasters, preparedness activities increase.^12^ In fact, signal detection theory^13^ would suggest that, given the frequency and magnitude of genuine threats, the belief that ‘it won't happen to us’ can actually reverse, and people become extremely vigilant about potential signals of danger. For example, in London in 2017, following a series of terrorist attacks earlier in the year, hundreds of people in Oxford Street fled in fear from a noise that turned out to be harmless.

The second factor is self-relevance. Self or identity exists at many levels, from personal identity (e.g. ‘I’ as an individual with unique personal characteristics) to the many social identities that are important to us based on our group memberships (e.g., national identity [‘us’ as British people’] or religious identity [‘us’ as Muslims]). The social categories we belong to can affect whether we perceive threats as relevant,^14^ and can facilitate either adherence to protective behaviours or the adoption of risky behaviours.^15^ If the threat of COVID-19 is understood principally in relation to the individual, then those individuals least at risk (those young and healthy) may well feel it is unnecessary to change their behaviours. Indeed, they might also feel they have a right to take risks with their own health.^16^ Thereby they continue to act in ways that put others at risk of infection. This perceived lack of self-relevance may be one reason why younger people have been among those with lower levels of adherence with public health regulations such as staying at home during ‘lockdown’.^17^ By contrast, where people perceive ‘us’ as being at risk, they are less likely to discount that risk.^14^

Most information about impending threat in a potential or actual emergency is indirect, or socially mediated: people do not see flames, rather they hear a fire alarm or they see others escaping. The third factor in perceiving threat is therefore communication about that threat, which needs to come from a trusted source. A source that provides too many false positives can lead to false negatives in behavioural response, as in the poor public response rate to fire alarm bells.^18^ Conversely a trusted source can convince there is a threat, even when it is novel and invisible. In the early weeks of the pandemic in the UK, trust in the government was high,^19^ as were public concerns about the threat of the virus^20^ (and those who denied the threat, for example, by drawing upon conspiracy theories or through mistrust of science,^21^ were in the minority).

Communication and hence influence regarding threat occurs not only through deliberate attempts (such as alarms and warnings), but also through observed examples of others’ conduct. What our peer groups, family, community and others appear to feel safe doing can serve to convey what it is safe for us to do. In all these cases, trustworthiness is linked to shared identity. When the source is seen as one of ‘us’, we are more likely to see their messaging and behaviour as relevant, more likely to listen, and more likely to follow their instructions or their example. This has been demonstrated in both ‘normal’ life^22^ and in relation to the COVID-19 pandemic.^23^

## Adherence to the required public health behaviours

Most of the required public health preventive behaviours during the COVID-19 pandemic involved some sacrifice of personal freedoms. Staying home and other aspects of lockdown were costly and onerous for most people, and much more for some groups than for others.^24^ Before lockdown in the UK, some doubted whether the public had the mental strength to endure these privations over time. This was most clearly expressed in early March by the Chief Medical Officer, who stated ‘There is a risk if we go too early people will understandably get fatigued and it will be difficult to sustain this over time’.^25^ This notion of ‘fatigue’ as a form of psychological frailty^4^ was quite separate from the expected stresses of quarantine^26^ and the mental health toll for many people (contrary to some claims, the notion of ‘behavioural fatigue’ was not suggested by behavioural scientists advising the UK Government; rather, those participating in SPI-B, the SAGE subgroup on behaviour, criticised the concept^25,27^). This contemporary concern with public mental fragility parallels government fears in the Second World War of public panic, shelter mentality and widespread breakdown in response to the Blitz.^28^ In both cases, there was a debate around the extent to which coercion would be needed to enforce public adherence.

Research on behaviour in other public health emergencies has shown that people would be willing to undergo prevention and mitigation measures at high personal cost, where these are perceived as legitimate public health interventions and where the relevant authority is respected and is seen as in-group (rather than as ‘other’). A good example is mass casualty decontamination, which is a procedure undertaken to remove contaminants from the skin of a potential casualty in the event of a chemical, biological, radiological or nuclear incident. Because the procedure can involve removing clothes in a public place, those affected may perceive decontamination as more threatening than the incident itself. Reviews of the literature find that any failure by responders to consider casualties’ psychosocial needs can lead to failures of the procedure, including people leaving the scene still contaminated and therefore endangering their families and communities.^29^ The reviews also showed that the use of coercive measures (threats, force, shouting) had a backfire effect rather than enhancing effective public engagement. However, when responders managing the procedure explained the importance of decontamination and provided regular updates about their actions, this increased perceptions of the legitimacy of the procedure among casualties. In turn, this enhanced shared identification between emergency responders and members of the public. Shared identification predicted: reduced public anxiety;^30^ greater public adherence with the procedure, cooperation,^30,31^ and greater speed and efficiency of the decontamination process.^32^ Subsequent research has demonstrated that effective engagement with the decontamination procedure is also increased when casualties perceive the effectiveness of the measures.^33^

Although staying at home and distancing more generally during lockdown were experienced as onerous, adherence rates were very high in the UK. (Adherence rates for self-isolation were much lower.^34^ As well as the commitment, this is likely to be because of material constraints, such as not having the financial support or support for shopping needs, and not understanding what self-isolation actually requires.^35,36^) This was the case on both behavioural measures^37^ and self-report measures across multiple sample.^19,38^ These high rates of adherence suggest that the public were indeed psychologically able to make the required sacrifices.^39^

A number of studies have now looked at predictors of this adherence. In addition to the perceived effectiveness of public health measures,^40^ many studies have identified group processes including social identification, similar to those evidenced in the decontamination research. Thus the Office for National Statistics data showed that at the same time that adherence rates were high during the strict lockdown, there was a strong sense among members of the public that people were becoming more united,^41^ suggesting a possible association between the two. The association between group processes, such as shared identification, and adherence to (or support for) public health measures, has now been demonstrated in cross-sectional and longitudinal survey studies as well as in experiments. Thus predictors include perceived norms within valued social groups,^42^ identification with one's community,^43^ sense of public duty,^44^ empathy with vulnerable groups,^45^ the belief that ‘we're all in it together’ (which was found to be more important than threats of coercion),^46^ social capital,^47^ national identification,^48^ horizontal collectivism^21^ and belief in shared values of security and responsibility.^49^ Overall, these factors evidence the importance of a sense of collectivity, at the group, community or national level, that has driven adherence behaviours.

There has been some shaming and stigmatising of those who are perceived not to be adhering.^50^ However, structural inequalities have been shown to affect ability to comply.^24^ In the same way that disadvantaged demographics are overrepresented in the infection and death figures,^25,51^ some groups have been less able to distance and were obliged to go into work on sometimes crowded trains, and were limited to busy public spaces when taking exercise.

As lockdown in the UK eased over May, June and July 2020, there was evidence that public adherence to the rules declined.^52,53^ Other developments over the same period suggest possible explanations for this decline more in line with the factors described above than behavioural fatigue.

First, the official messaging changed. In May, the official slogan ‘stay at home’ was replaced with ‘stay alert’, shifting from a clear behavioural imperative to a somewhat vaguer instruction on how to feel. There were also a large number of government announcements on changes to the rules in May, June and July. There is some evidence that official communications were becoming less effective both in communicating risk and in providing information on mitigations. Over this period, perceptions of being unsafe reduced^54^ and public knowledge of the rules declined.^55^ Further, the most significant relaxation of lockdown – which included being allowed to visit a pub, cafe or restaurant, museum, library, theme park, cinema, hotel, hairdresser or barber – that took place on 4 July, appeared to have a strong signalling effect (not least, perhaps, because these were heralded in the mass media as ‘freedom’ and as the ‘end of lockdown’). Thus, surveys report drops in people's reported adherence to physical distancing shortly afterward.^53,56^

Second, there were some significant changes in terms of national unity and the public's relationship with the government that interacted with these problems with communication. A number of sources show that the sense of national unity that was evident at the start of the pandemic declined over time.^57^ The notion that ‘we are all in it together’ was flatly contradicted by the evidence that structural differences between groups – the greater economic losses in terms of jobs and pay for some and not others, and the mounting evidence of particular risk among ethnic minorities^51^ – was being exacerbated by the pandemic.

A significant feature of this decline in a sense of national unity was a loss of trust in the UK Government. In particular, lack of government repentance over the actions of one special advisor, who admitted breaking the rules on lockdown, served to communicate that there were one set of rules for the privileged and another set for everyone else who had made the sacrifices of staying at home and not visiting family; this decline in trust was in turn associated with a drop-off in adherence.^58^ (Other research^59^ suggests that there were two types of public response to this incident. First, there was cynicism about the rules, which was associated with lower levels of physical distancing adherence. Second, those who were angry about the actions of the government advisor were less likely than others to believe that it was acceptable to bend the rules, but more likely than others to comply with the guidelines themselves. This latter pattern is in line with Stephen Reicher's suggestion that the advisor would be seen as an anti-role model.^60^)

## Neighbourhood support and community mutual aid groups

Government policies such as the community resilience programme^61,62^ are a recognition that public involvement, in the form of active support for others affected, is a necessary part of emergency response. (Although governments started officially acknowledging this after 9/11,^63^ some disadvantaged groups have long made their own resilience plans in the knowledge that the state will not provide them with the support and resources they need in times of crisis.^64^) Research on emergencies and disasters shows that such public involvement is common.^65^ Of course, not every member of the public provides support; and some emergency events evidence more support behaviours from the public than others. But more lives are saved by the ‘average’ citizen, whether ‘bystander’ or fellow survivor, than are saved by professionals.^5^

How does this public support come about? Research on a variety of emergencies and disasters suggests that an underlying mechanism is that of a shared social identity among those affected.^65^ Shared social identity facilitates support in three ways. It motivates people to give support to others (since their interest is seen as ‘our’ interest).^65^ It increases expected support within the in-group (and hence enables a coordinated response).^66^ And it means that offers of help are perceived as genuine rather than reflecting ulterior motives (which means that the help is more likely to be accepted).^67^ Most group identities reflect long-standing group memberships; hence, existing social capital is one of the major factors in community resilience.^68^ But an emergency can also create a completely new group identity or sense of ‘we-ness’ among those affected, based on common fate.^69^ Further, in this context, the supportive behaviour of fellow in-group members can serve to define supportive group norms, and identification with the group increases the influence of such norms.^70^

At the start of the COVID-19 pandemic, there was a massive upsurge in mutual caring behaviours among members of the public.^71^ Mutual social support among members of the public took different forms, partly reflecting community members’ differing needs. Moreover, the requirement for some to self-isolate at home meant that there were multiple simultaneous needs. Neighbours helped neighbours with shopping, collecting medical prescriptions, dog walking, providing information and emotional support.^72^ For example, in one week in April 2020, the Office for National Statistics survey found that nearly two-thirds of adults surveyed said other local community members would support them if they needed help during the pandemic, and over one in three adults said they had done shopping or other tasks for neighbours in the previous week.^73^ Identification with the community has been shown to be a predictor of helping neighbours in these ways.^43^

In addition to informal interactions between neighbours, a very large number of organised mutual aid and community support groups were set up. One estimate made in May 2020 was that there were 4300 such groups connecting an estimated 3 million people in the UK.^74^ These ranged from Facebook groups (which acted simply to connect requests for help with offers from volunteers) to those groups that cooked food and carried out deliveries themselves. One research study concluded that such groups have been crucial in the society's response to the pandemic.^75^

A number of studies are now investigating the psychology of volunteering and mutual aid during the pandemic. A survey by Abrams et al^76^ found that, compared with other participants, those who had volunteered to help others in the context of the pandemic reported higher trust in others to follow the guidelines, higher trust in the government, higher compassion for people living in their local area and stronger connections with their family, friends, colleagues and neighbours. Although some mutual aid groups have been ‘emergent’, reflecting new relationships among participants,^75^ many others are extensions of existing community groups and are based on existing social capital.^75^

Previous research on ‘altruistic communities’ in other kinds of disaster^77^ suggests that these groups decline over time as people run out of emotional and physical resources or the state steps in.^78^ There has been only limited research on how such groups sustain themselves over time. But it would seem that some of the factors that can help include a group identity, a place to meet and talk about the group and its aims, commemorative events, support (but not co-option) from local authorities and alliances with other groups.^5,79^

## Conclusions and recommendations

There are many reasons why it is important to understand the role of group processes in the COVID-19 pandemic, but we will focus here on two of the most important ones. The first reason is that, without a proper understanding of group processes, practitioners and policy makers might instead make decisions by drawing upon ‘folk psychologies’. Examples of folk psychology include the assumption that the public will necessarily panic and be incapable of taking the responsibility needed when crisis strikes.^7^ Emergency management strategies based on these assumptions, such as use of coercion and withholding information from the public, are known to be ineffective and even counterproductive.^5^ In the case of COVID-19, we have just seen the damaging consequences of relying on folk-psychological assumptions. The ‘common sense’ notion of supposed public fatigue was one explanation given for the timing of the implementation of necessary physical distancing and ‘lockdown' measures, with critics suggesting that the implementation of such measures was delayed and that this delay has cost lives.^52,80^

Second, if behaviour in the pandemic is a function of mutable group processes rather than fixed tendencies, then behavioural change is possible. This means that we can help design more effective interventions to facilitate collective resilience in the face of the pandemic and other collective threats. Therefore, based on the above, and in line with other sets of recommendations, both in relation to the pandemic^5,81–83^ and to emergencies more generally,^2,84^ our recommendations are as follows.

*Provide clear and credible information on risk from a trusted source*. People are more persuaded by messages from fellow in-group members than out-group members. More specifically, where a group member is seen as embodying the group's identity and values, this increases follower adherence to their guidance.^85^ Therefore, those responsible for the communication of information on risk need to ensure that the messaging is clear, but also to consider who would be the best person to deliver that message and how that person can create a connection with the audience.

*Focus on our common interests and identity to engage people in the commitments that need to be made (which are mostly for others rather than for the individual)*. The importance of clear messaging extends from the nature and extent of the threat to the actions that people need to take. The UK Government messaging that justified staying at home in terms of ‘protecting the NHS’, and that promoted distancing behaviours and wearing face coverings for others (rather than the personal self), were some of the most effective.

*Listen to, recognise, and resource community support groups to give them agency to sustain themselves over time.* In the UK, the importance of community mutual aid groups is recognised at a national level by the Civil Contingencies Secretariat and the Communities Prepared National Group, as well as by many local resilience forums. It is evident that official bodies have learned a lot from mutual aid groups. There is understanding that such groups play a vital role and need to be supported.^75^ But it also important that support for such groups is based on the group's needs even if these do not completely align with those of the authorities.

At the time of writing (October 2020), we are still in the relatively early days of the pandemic. Although many research studies have been carried out on public behaviour during the pandemic, there is still much we do not know. Priorities for the future include understanding how the group processes described here can contribute to recovery, mitigate the ongoing effects of secondary stressors on mental health, and enhance public engagement with vaccines.

## Data Availability

Data availability is not applicable to this article as no new data were created or analysed in this study.
